# The complete chloroplast genome sequence of the medicinal plant *Gonostegia hirta* (Bl.) Miq. (Urticaceae)

**DOI:** 10.1080/23802359.2020.1823262

**Published:** 2020-09-18

**Authors:** Feiya Zhao, Aien Tao, Jinfu Qian

**Affiliations:** School of Medicine, Tourism and Culture College of Yunnan University, Lijiang, China

**Keywords:** *Gonostegia hirta*, chloroplast, Illumina sequencing, phylogeny

## Abstract

The total length of the chloroplast genome was 159,086 bp, with 35.9% overall GC content and exhibited typical quadripartite structure, a pair of IRs (inverted repeats) of 30,727 bp was separated by a small single copy (SSC) region of 18,661 bp and a large single copy (LSC) region of 78,971 bp. The cp genome contained 107 genes, including 77 protein-coding genes, 30 tRNA genes, and four rRNA genes. The phylogenetic analysis indicated Gonostegia was closely related to Debregeasia

*Gonostegia* is a genus of the Urticaceae family, which includes 12 species in the world. Most of them are widespread in Asia, Australia. *Gonostegia hirta* is an ethnic medicine commonly used as the treatment of Stomachache, Bleeding, Mastitis (Minru and Yi 2016). However, up to now for such medicinal plant, many studies have focused on describing its chemical compositions (Jun et al. 2013; He-Dong et al. 2014), However with little involvement in its genomes, Here, we report the chloroplast genome sequence of *G. hirta* and find its internal relationships within the family Urticaceae.

Fresh and clean leave materials of *G. hirta* were collected from Lijiang city, Yunnan, China (N27°00′44.48″/E100°14′42.42″), and the plant materials and a voucher specimen (TC31) were deposited at Tourism and Culture College of Yunnan University (Lijiang).Total genomic DNA was extracted using the improved CTAB method (Doyle 1987; Yang et al. 2014), and sequenced with Illumina Hiseq 2500 (Novogene, Tianjing, China) platform with pair-end (2 × 300 bp) library.About5.19 Gb of raw readswith17,290,260 paired-end reads were obtained from high-throughput sequencing. The raw data was filtered using Trimmomatic v.0.32with default settings (Bolger et al. 2014). Then paired-end reads of clean data were assembled into circular contigs using GetOrganelle.py (Jin et al. 2018) with *Debregeasia orientalis* (NC_041413) as reference. Finally, the plastome was annotated by the PGA (Plastid Genome Annotator) (Qu et al. 2019) with manual adjustment using Geneious v. 7.1.3 (Kearse et al. 2012). Then the annotated chloroplast genome was submitted to the GenBank under the accession number MT012416.

The complete cp genome sequence of *G.Hirta* was 159,086 bp in length, with a large single-copy region (LSC) of 78,971 bp, a small single-copy region (SSC) of 18,661 bp, and a pair of inverted repeats (IR) regions of 30,727 bp. A total of 107 genes were annotated, including 77 protein-coding genes, 30 tRNA genes, and four rRNA genes. The GC content of the cp genome is 35.9%. To reveal the phylogenetic position of *G.Hirta* with other members in Urticaceae, a phylogenetic analysis was performed based on 6 complete cp genomes, and taxa from Rubiaceae, *Morinda citrifolia* (MN699649), *Morinda citrifolia* (NC 047302), *Hedyotis ovata* (MK203877), *Rubia cordifolia* (MN736957), *Rubia cordifolia* (NC 047470), *Gynochthodes nanlingensis* (NC 028614) were served as outgroups. The sequences were aligned by MAFFT v7.309 (Katoh and Standley 2013). The neighbor-joining (NJ) tree analysis was conducted using MEGA v.7.0.26 (Kumar et al. 2016) with 1000 bootstrap replicates. The phylogenetic result showed that Gonostegia was closely related to Debregeasia (Figure 1). Meanwhile, the phylogenetic relationship in Urticaceae was consistent with previous studies and this will be useful data for developing markers for further studies.

**Figure 1.**
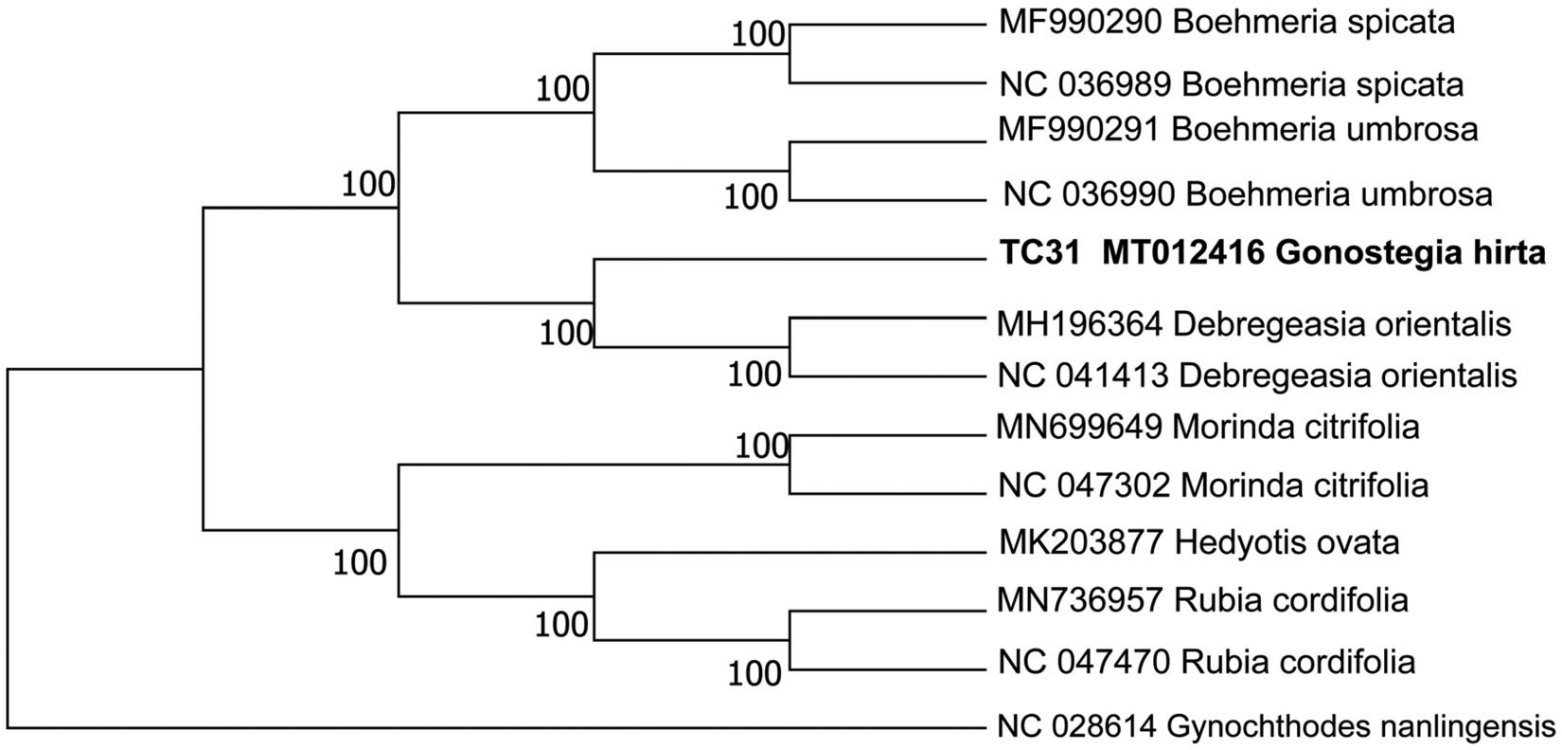
Phylogenetic position of *G. hirta* inferred by neighbor-joining (NJ) tree of complete cp genomes.

## Data Availability

The data that support the findings of this study are openly available in GenBank of NCBI at https://www.ncbi.nlm.nih.gov, reference number MT012416.
